# Integrated RNA Sequencing and Multi‐Bioinformatics Analyses Reveal the Molecular Mechanisms Underlying the Anti‐Breast Cancer Effects of the Fritillariae Thunbergii Bulbus–Bolbostemmatis Rhizoma Herb Pair

**DOI:** 10.1002/cbf.70285

**Published:** 2026-08-26

**Authors:** Quan Tang, Dong Niu, He Li, Heng Zhou Zhu, Chun Hui Jin, Guo Qing Zhao, Xiao Dan Zhu

**Affiliations:** ^1^ Department of Oncology Nanjing University of Chinese Medicine Wuxi Affiliated Hospital: Wuxi Hospital of Traditional Chinese Medicine Wuxi P.R. China; ^2^ Office of Academic Research, Nanjing University of Chinese Medicine Wuxi Affiliated Hospital: Wuxi Hospital of Traditional Chinese Medicine Wuxi P.R. China

**Keywords:** bioinformatics, breast cancer, EPCAM, Fritillariae Thunbergii Bulbus–Bolbostemmatis Rhizoma, machine learning

## Abstract

Breast cancer (BC) is a highly heterogeneous malignancy with complex molecular mechanisms, highlighting the need for effective biomarkers and therapeutic targets. The Fritillariae Thunbergii Bulbus–Bolbostemmatis Rhizoma (ZBM–TBM) herb pair has been used in the treatment of BC, although its potential molecular mechanisms remain incompletely understood. In this study, an integrated strategy combining transcriptomic analysis, machine learning, liquid chromatography–tandem mass spectrometry (LC–MS/MS), network pharmacology, molecular docking, molecular dynamics (MD) simulations, and single‐cell virtual knockout analysis was employed to identify BC‐associated molecular biomarkers and explore the potential mechanisms underlying the effects of ZBM–TBM. Integration of weighted gene co‐expression network analysis with least absolute shrinkage and selection operator regression, random forest, and support vector machine–recursive feature elimination identified EPCAM as the only common core gene. EPCAM was significantly upregulated in BC tissues and showed favorable diagnostic performance (AUC = 0.903), while high EPCAM expression was associated with shorter overall survival (log‐rank P = 0.032). Functional analyses indicated that EPCAM was associated with tumor‐related and immune‐related biological processes, including cell cycle regulation, DNA replication, and immune regulation. LC–MS/MS analysis identified 32 major active constituents of ZBM–TBM, and subsequent network pharmacology analysis identified 33 overlapping targets and 10 hub genes enriched in multiple tumor‐ and immune‐related signaling pathways, including the Notch, IL‐17, HIF‐1, and TGF‐β signaling pathways. Although ZBM–TBM did not directly target EPCAM in the predicted target network, computational analyses indicated functional associations between EPCAM and hub gene‐associated regulatory networks. Molecular docking and MD simulations further suggested that peiminine may represent a potential active constituent, exhibiting favorable predicted binding characteristics with multiple candidate targets, particularly AURKA. Single‐cell virtual knockout analysis showed that computational perturbation of AURKA was associated with alterations in cell cycle‐, extracellular matrix‐, and cell adhesion‐related processes, supporting its potential functional relevance at the cellular level.

**Abbreviations**
ATM: Ataxia‐telangiectasia mutated
AUC: Area under the curve
BC: Breast cancer
CIBERSORT: Cell‐type Identification By Estimating Relative Subsets Of RNA Transcripts
ECM: Extracellular matrix
EMT: Epithelial–mesenchymal transition
GAFF: General Amber force field
GEO: Gene Expression Omnibus
GO: Gene Ontology
GSEA: Gene Set Enrichment Analysis
IL‐17: Interleukin‐17
KEGG: Kyoto Encyclopedia of Genes and Genomes
LASSO: Least absolute shrinkage and selection operator
LC–MS/MS: Liquid chromatography–tandem mass spectrometry
MD: Molecular dynamics
MM/GBSA: Molecular mechanics/generalized Born surface area
PCA: Principal component analysis
PDB: Protein Data Bank
PPI: Protein–protein interaction
RESP: Restrained electrostatic potential
RF: Random forest
Rg: Radius of gyration
RMSD: Root mean square deviation
RMSF: Root mean square fluctuation
RNA‐seq: RNA sequencing
ROC: Receiver operating characteristic
SNN: Shared nearest neighbor
SVM‐RFE: Support vector machine‐recursive feature elimination
TBM: Bolbostemmatis Rhizoma
TCGA‐BRCA: The Cancer Genome Atlas‐Breast Cancer
TCM: Traditional Chinese medicine
TF: Transcription factor
TGF‐β: Transforming growth factor‐beta
Tfh: Follicular helper T cell
Treg: Regulatory T cell
t‐SNE: t‐distributed stochastic neighbor embedding
WGCNA: Weighted gene co‐expression network analysis
ZBM: Fritillariae Thunbergii Bulbus

## 1 Introduction

Breast cancer (BC) is one of the most common malignant tumors among women worldwide and is characterized by marked heterogeneity and complex molecular mechanisms. Its incidence has continued to increase annually, making it a major threat to global women's health [1, 2]. Although modern therapeutic strategies, including surgery, radiotherapy, chemotherapy, and targeted therapy, have achieved considerable progress in the treatment of BC, these approaches are still associated with tumor recurrence, drug resistance, and severe adverse effects, all of which substantially impair patients' quality of life and prognosis [3]. Therefore, further investigation into the molecular pathogenesis of BC, along with the identification of novel biomarkers and therapeutic targets, is urgently needed to develop safer and more effective comprehensive treatment strategies.

With the rapid development of bioinformatics, machine learning approaches have provided powerful tools for drug target screening and mechanistic investigations [4]. By integrating weighted gene co‐expression network analysis (WGCNA) with multiple machine learning algorithms, including least absolute shrinkage and selection operator (LASSO) regression, random forest (RF), and support vector machine recursive feature elimination (SVM‐RFE), key genes associated with BC can be efficiently identified from large‐scale transcriptomic datasets, thereby facilitating the discovery of potential biomarkers. In the present study, multiple algorithms were integrated to identify key genes closely associated with BC, particularly EPCAM, followed by exploration of its potential molecular functions and clinical significance. However, the role of EPCAM within complex regulatory networks, especially its relationship with pharmacological interventions, remains to be further elucidated.

Traditional Chinese medicine (TCM), supported by thousands of years of clinical practice, has demonstrated unique advantages in the treatment of tumors, including BC. In addition to exerting direct antitumor effects, TCM can also be combined with chemotherapy and targeted therapies to achieve synergistic effects by enhancing efficacy while reducing toxicity [5, 6]. The herb pair consisting of Fritillariae Thunbergii Bulbus (ZBM) and Bolbostemmatis Rhizoma (TBM) has been widely used in the treatment of BC and various other tumors [7]. ZBM, derived from the bulbs of plants belonging to the Liliaceae family, possesses the pharmacological properties of clearing heat, resolving phlegm, and dissipating nodules, with its major active constituents including alkaloids, polysaccharides, and total saponins [8]. TBM, derived from the pseudobulbs of plants in the genera Cremastra or Pleione, exhibits functions associated with heat‐clearing, detoxification, and dissipation of swelling and masses [9]. Previous studies have demonstrated that the combined application of ZBM and TBM exerts synergistic effects in BC treatment by enhancing mass‐dissipating activity, alleviating local symptoms such as redness, swelling, heat, and pain, and suppressing tumor growth [10]. Modern pharmacological studies have further shown that the major constituents of ZBM and TBM possess diverse biological activities, including anti‐ulcer, analgesic, anti‐inflammatory, antioxidant, antiproliferative, and multidrug resistance‐reversing effects [9, 11]. For example, peimine and peiminine have been reported to significantly reverse multidrug resistance in the MCF‐7/ADR BC cell line, thereby enhancing therapeutic efficacy [12].

Although the ZBM–TBM herb pair has demonstrated therapeutic efficacy in the clinical management of BC, the key active constituents and precise molecular mechanisms underlying its antitumor effects have not yet been fully elucidated, which to some extent limits its broader application and further development. Given the limitations of conventional network pharmacology in active compound screening and target prediction [13], the present study employed liquid chromatography–tandem mass spectrometry (LC–MS/MS) to systematically characterize the chemical constituents of ZBM–TBM and constructed a network pharmacology framework based on transcriptomic data to identify its key active compounds and therapeutic targets. Furthermore, to comprehensively elucidate its mechanisms at both molecular and cellular levels, molecular docking and molecular dynamics (MD) simulations were performed to evaluate the binding characteristics between active compounds and target proteins, while single‐cell virtual knockout analysis was conducted to investigate the potential regulatory functions of key genes in cellular processes.

Therefore, this study aimed to employ an integrated research strategy combining machine learning and multiple bioinformatics approaches to identify key molecular biomarkers associated with BC and explore the potential molecular mechanisms underlying the effects of the ZBM–TBM herb pair in BC treatment. The findings are expected to provide novel computational insights and theoretical support for precision therapy and individualized medication in BC, while also promoting the innovative application of TCM in modern oncology.

## 2 Methods

### 2.1 Data Sources and Preprocessing

The transcriptomic dataset GSE15852 was obtained from the Gene Expression Omnibus (GEO) database. The dataset included 43 paired BC tissues and adjacent non‐tumor breast tissues (normal tissues) (*n* = 86 in total). Gene expression profiles were standardized and annotated based on the original platform annotation files to construct a normalized expression matrix with samples as rows and gene symbols as columns. Corresponding clinicopathological information was integrated to support downstream analyses.

### 2.2 WGCNA

WGCNA was performed based on the GSE15852 dataset to identify BC‐related functional modules and key genes. First, the raw expression matrix was subjected to log2 transformation to normalize the data distribution. Hierarchical clustering analysis was then conducted to detect and remove potential outlier samples, thereby improving the robustness of network construction. Subsequently, the optimal soft‐thresholding power was determined by evaluating the scale‐free topology fit index (R^2^) and mean connectivity under different threshold values. A weighted co‐expression network and topological overlap matrix were then constructed. Initial gene modules were identified using the dynamic tree‐cutting algorithm, followed by merging of highly similar modules through module eigengene clustering to obtain the final stable co‐expression module set. Pearson correlation analysis between module eigengenes and tumor/normal phenotypes was subsequently performed to identify modules significantly associated with BC. Finally, the relationships between module membership and gene significance within candidate modules were evaluated to identify potential hub regulatory genes. All analyses were performed in the R 4.3.1 environment using the limma and WGCNA packages.

### 2.3 Differentially Expressed Gene Screening and Integration

Three machine learning algorithms, including LASSO regression, RF, and SVM‐RFE, were comprehensively applied for candidate gene screening. LASSO regression was conducted using the glmnet package in R with 10‐fold cross‐validation, and the optimal penalty parameter λ.min was selected to obtain the feature gene set. The RF model was constructed using the randomForest package with 180 decision trees, and genes were ranked according to importance scores. SVM‐RFE was implemented using the e1071 and caret packages to recursively eliminate features. Cross‐validation was used to evaluate the classification performance of different feature subsets and determine the optimal number of features. Subsequently, the screening results from the three machine learning algorithms and WGCNA were integrated. Candidate gene sets identified by each method were extracted, and an UpSet plot was generated to visualize the union and overlap among gene sets. Furthermore, the intersection of the four methods was analyzed, and a Venn diagram was constructed to identify high‐confidence core genes shared by all approaches. Set calculations and visualization analyses were performed in R 4.3.1 using the UpSetR and VennDiagram packages.

### 2.4 Gene Set Enrichment Analysis (GSEA)

GSEA was performed based on transcriptomic expression data. Spearman correlation coefficients between EPCAM and all genes across the genome were first calculated, and genes were ranked according to correlation coefficients to generate a ranked gene list. Enrichment analyses were subsequently performed using four gene set collections, including Kyoto Encyclopedia of Genes and Genomes (KEGG), Gene Ontology (GO), oncogenic signatures, and immunologic signatures. GSEA was conducted using the clusterProfiler package (v4.0.5) in R 4.3.1. Enrichment results were visualized using the enrichplot, ggplot2, and cowplot packages.

### 2.5 Evaluation of Diagnostic Performance and Survival Analysis

RNA sequencing data and matched clinical follow‐up information from the TCGA‐BRCA cohort were used to evaluate the diagnostic and prognostic values of candidate genes. Strictly screened breast invasive carcinoma samples were included after exclusion of normal tissues and samples lacking clinical information. Expression data were normalized using log_2_(TPM + 1). For diagnostic analysis, the Mann–Whitney U test was used to compare gene expression differences between tumor and normal tissues, while receiver operating characteristic (ROC) curves were used to calculate the AUC and corresponding 95% confidence intervals (95% CI). For survival analysis, the optimal cutoff value was determined using the survminer::surv_cutpoint() function to classify patients into high‐ and low‐risk groups. Kaplan–Meier survival analysis combined with the log‐rank test was used to evaluate survival differences between groups. The proportional hazards assumption was verified using Schoenfeld residual tests (*p* > 0.05). All analyses were conducted in R 4.3.1 using the survival, survminer, pROC, and ggplot2 packages.

### 2.6 LC–MS/MS Analysis

ZBM and TBM herbal materials were provided by the Traditional Chinese Medicine Pharmacy of Wuxi Hospital of Traditional Chinese Medicine, with a total mass of 105 g. After soaking for 30 min, 1050 mL of purified water was added, and the mixture was decocted at slight boiling for 1 h to obtain the first extract. The residue was then extracted again with 840 mL purified water under the same conditions for 40 min to obtain the second extract. The two extracts were combined, homogenized thoroughly, aliquoted into freeze‐drying trays, sealed, and frozen at −80°C for 12 h. Samples were subsequently transferred into a freeze dryer pre‐cooled to −40°C, followed by vacuum freeze‐drying for 24 h. The resulting lyophilized samples were sealed and stored under dry conditions for subsequent analyses.

LC–MS/MS was performed for qualitative analysis of ZBM and TBM samples. Samples were accurately weighed and dissolved in 50% methanol to prepare 10 mg/mL solutions, vortex‐mixed, ultrasonically extracted for 30 min, and centrifuged at 14,000 rpm at 4°C for 10 min. The supernatants were collected for analysis. Chromatographic separation was carried out using a Vanquish ultra‐high‐performance liquid chromatography system (Thermo Fisher Scientific, USA) equipped with an ACQUITY UPLC HSS T3 column (2.1 × 100 mm, 1.8μm). The mobile phase consisted of acetonitrile (B) and 0.1% formic acid aqueous solution (A), using the following gradient elution program: 0–24 min, 5%–100% B; 24–26.5 min, 100% B; 26.5–27 min, 100%–5% B; and 27–30 min, 5% B. The column temperature was maintained at 35°C, the flow rate was 0.3 mL/min, and the injection volume was 2 μL.

Mass spectrometric detection was performed using an Orbitrap Exploris 240 mass spectrometer (Thermo Fisher Scientific, USA) equipped with a heated electrospray ionization source. Spray voltages were set to +3.5 kV and −3.0 kV in positive and negative ion modes, respectively. The ion transfer tube temperature was maintained at 320°C, and the vaporizer temperature was set at 350°C. Sheath gas and auxiliary gas flow rates were 35 and 10 L/h, respectively. Full MS scans were acquired at a resolution of 60,000 over an m/z range of 100–1500, followed by data‐dependent MS/MS acquisition of the top five most intense ions at a resolution of 15,000 using collision energies of 10, 30, and 50 eV. Data acquisition was performed using Xcalibur software, while Compound Discoverer software was used for data preprocessing and compound identification.

### 2.7 Identification of Common Target Genes between ZBM–TBM and BC

Based on the LC–MS/MS identification results, candidate active constituents of ZBM–TBM were obtained after classification, visualization, and removal of low‐abundance irrelevant variables. The identified compounds were searched in the PubChem database to obtain their corresponding simplified molecular‐input line‐entry system (SMILES) structures. Subsequently, the SMILES structures were imported into the SwissTargetPrediction database with the species restricted to Homo sapiens for potential target prediction. Target genes with prediction probabilities greater than 0.1 were retained, and redundant targets were removed to generate the potential target set corresponding to the active constituents. BC‐related genes were obtained from the union of genes identified by the four screening approaches. Intersection analysis was then performed between the predicted drug targets and BC‐related genes, followed by construction of a Venn diagram to visualize the number of overlapping genes. Finally, Cytoscape software (v3.9.1) was used to construct a ZBM–TBM–BC–target gene interaction network to illustrate candidate therapeutic targets.

### 2.8 GO and KEGG Enrichment Analyses of Common Target Genes

To investigate the biological functions of the overlapping genes, GO and KEGG enrichment analyses were performed using the Metascape database. GO analysis was conducted to predict biological processes, cellular components, and molecular functions, whereas KEGG pathway analysis was used to identify significantly enriched signaling pathways. The GO and KEGG enrichment results were subsequently imported into R 4.3.1 for visualization and further analyses.

### 2.9 Immune Infiltration and Correlation Analyses

The CIBERSORT algorithm was first applied to estimate the relative proportions of 22 immune cell subtypes within the samples. RNA sequencing expression matrices from the TCGA‐BRCA cohort were used as input data. Immune cell proportion analyses were conducted in the R 4.3.1 environment and visualized using the ggplot2 and pheatmap packages. Differences in immune cell proportions between tumor and normal groups were assessed using the Mann–Whitney U test, with a two‐sided *p* < 0.05 considered statistically significant. Correlations among immune cell subtypes were also analyzed to evaluate synergistic and antagonistic relationships between different immune populations. Subsequently, Spearman correlation analysis was performed between the estimated immune cell proportions and the expression levels of the 10 identified hub genes. All analyses were performed in R 4.3.1.

### 2.10 Construction of the TF–mRNA–miRNA Regulatory Network

A transcription factor (TF)–messenger RNA (mRNA)–microRNA (miRNA) regulatory network was constructed by integrating TF–mRNA and miRNA–mRNA interactions. Experimentally validated TF–target regulatory pairs were obtained from the TRRUST database (v2.0), while miRNA–target interactions were retrieved from the miRWalk database (v3.0). For each hub gene, the top five miRNA interactions with the lowest binding energies were selected. The regulatory interactions were integrated using shared mRNAs as bridges, and Cytoscape software (v3.9.1) was used to construct and visualize the interaction network. This network was subsequently analyzed to identify key hub nodes as well as potential feedback and feedforward regulatory loops.

### 2.11 Construction of the Gene Co‐Occurrence Network

Gene expression correlation analysis was conducted based on the GSE15852 expression matrix in the R 4.3.1 environment. The cor_test function from the correlation package was used to calculate Pearson correlation coefficients and corresponding significance levels with 95% confidence intervals. Global pairwise correlations within the expression matrix were evaluated using the correlation() function. Scatter plots were generated using the ggplot2 and ggpubr packages for visualization.

### 2.12 Gene Expression Correlation Analysis

Based on the GSE15852 expression matrix, gene expression correlation analysis was performed in R 4.2.1. The cor_test function from the correlation package was used to calculate Pearson correlation coefficients with 95% confidence intervals, assessing variable relationships and significance levels. Global pairwise correlations within the matrix were evaluated using the correlation() function. Scatter plots were generated for visualization using ggplot2 and ggpubr packages.

### 2.13 Molecular Docking and Visualization

A drug–active constituent–target gene interaction network was constructed based on candidate active compounds identified by LC–MS/MS analysis and their predicted target genes. AutoDock Vina 1.2.2 software was used to evaluate the binding modes and binding energies between core active compounds and corresponding targets. Molecular structure files of active compounds were downloaded from the PubChem database, while three‐dimensional protein structures were obtained from the Protein Data Bank (PDB). Protein and ligand structures were preprocessed by removing water molecules, adding polar hydrogen atoms, and converting all files into PDBQT format. Grid boxes were then positioned around the active regions of target proteins to ensure coverage of potential binding pockets with appropriate search spaces. Docking calculations were subsequently performed using AutoDock Vina, and binding energies were visualized using bubble plots to illustrate affinities between compounds and targets. Finally, docking conformations were visualized using PyMOL software to present ligand conformations and interaction patterns within protein binding pockets.

### 2.14 Molecular Dynamics Simulation

MD simulations were performed using Gromacs 2022.3 software. Small molecules were preprocessed using AmberTools22 with the general Amber force field (GAFF), while Gaussian 16 W was used for hydrogen addition and restrained electrostatic potential (RESP) charge calculations. The calculated electrostatic potential data were incorporated into the topology files of the MD systems. Simulations were conducted at a constant temperature of 300 K and pressure of 1 bar using the Amber99sb‐ildn force field. The systems were solvated using the TIP3P water model, and appropriate numbers of Na^+^ ions were added to neutralize the total system charge.

Energy minimization was first performed using the steepest descent algorithm, followed by equilibration under constant volume and temperature (NVT) and constant pressure and temperature (NPT) ensembles for 100,000 steps each. The coupling constant was set to 0.1 ps, and equilibration lasted for 100 ps. Subsequently, a 100 ns production MD simulation was carried out with a total of 5,000,000 steps and a time step of 2 fs. After simulation completion, trajectory analyses were conducted using built‐in Gromacs tools to calculate root mean square deviation (RMSD), root mean square fluctuation (RMSF), radius of gyration (Rg), molecular mechanics/generalized Born surface area (MM/GBSA) binding free energy, and free energy landscape profiles.

### 2.15 Single‐Cell Virtual Knockout Analysis

Single‐cell RNA sequencing data from the GSE268662 dataset in the GEO database were used for analysis. Raw expression matrices were imported into R 4.3.1 and processed using the Seurat package (v4.3.0). Quality control was first performed to remove low‐quality cells, followed by data normalization and identification of highly variable genes.

Principal component analysis (PCA) was used for preliminary dimensionality reduction, followed by visualization using t‐distributed stochastic neighbor embedding (t‐SNE). Unsupervised clustering was performed based on the shared nearest neighbor (SNN) algorithm, and cell clusters were annotated using canonical marker genes. To evaluate the functional role of AURKA at the single‐cell level, a virtual knockout strategy was employed to computationally perturb AURKA expression, and network‐based analysis was performed to infer its effects on global transcriptomic profiles. Differentially expressed genes before and after perturbation were identified using an adjusted *p* value < 0.05 as the screening criterion. GO and KEGG enrichment analyses were subsequently performed in R to investigate the biological functions associated with perturbation‐related genes.

## 3 Results

### 3.1 WGCNA and Module Detection

**Figure 1:**
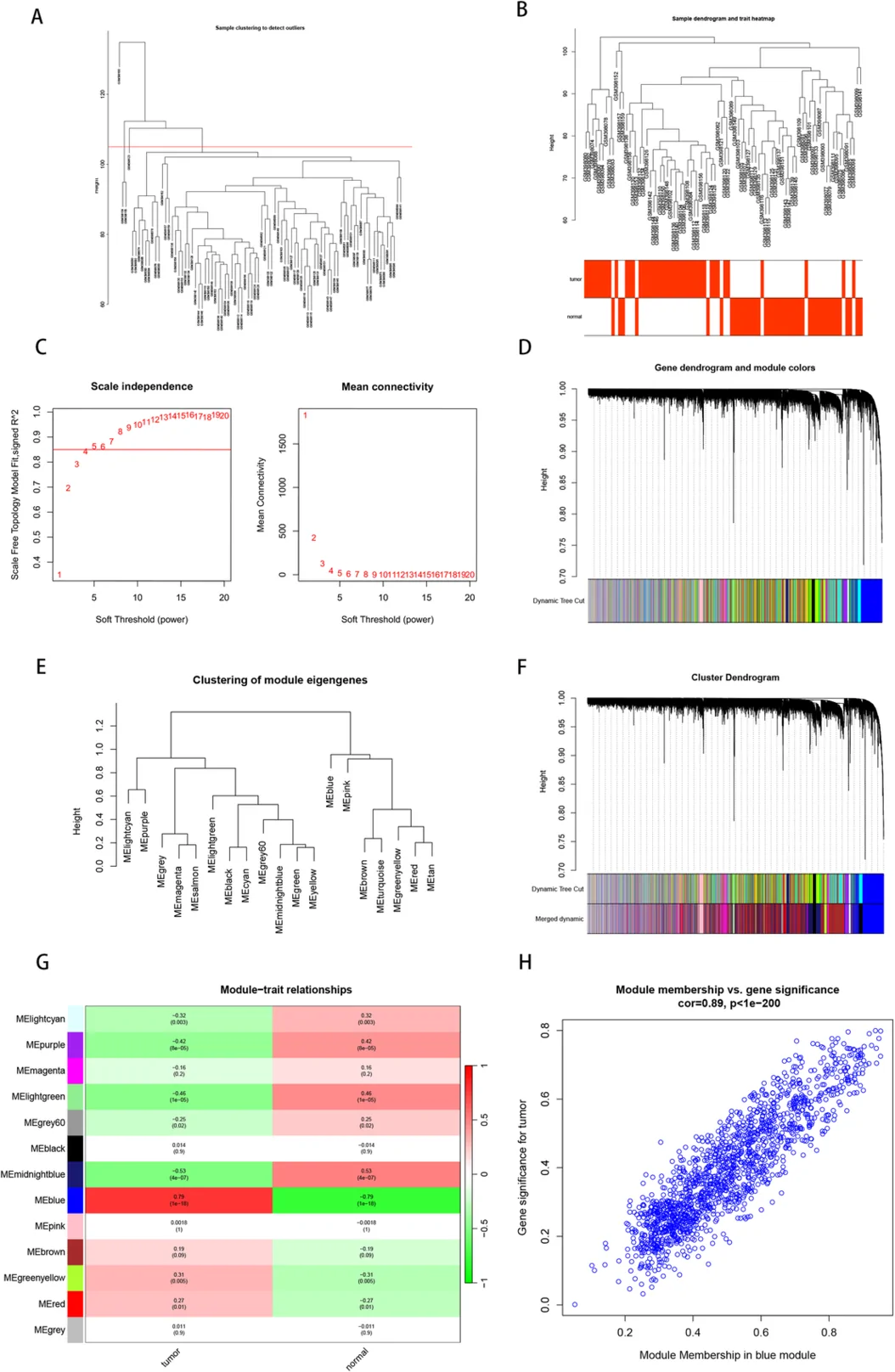
WGCNA and Module Detection. A: Hierarchical clustering dendrogram of samples used for identification and removal of outlier samples. B: Sample clustering dendrogram combined with the tumor/normal phenotype heatmap. C: Soft‐threshold selection analysis. The left panel shows the scale‐free topology fit index under different β values, and the right panel shows the mean connectivity curve. β = 8 was selected for network construction. D: Gene clustering dendrogram and initial module assignment, with different colors representing distinct co‐expression modules. E: Clustering dendrogram of module eigengenes showing similarities among module expression patterns. F: Gene clustering dendrogram and module assignment after merging highly similar modules. G: Heatmap showing correlations between modules and the BC phenotype. Values represent Pearson correlation coefficients and corresponding P values. H: Scatter plot showing the relationship between module membership and gene significance within the MEblue module.

To identify gene modules associated with BC, WGCNA was performed using the GSE15852 dataset, resulting in the identification of 643 feature genes (Figure 1). Sample clustering analysis revealed the presence of outlier samples, and a cut height of 105 was applied to remove obvious outliers. Soft‐threshold analysis demonstrated that when β = 8, the scale‐free topology fit index (R^2^) reached 0.90 while maintaining moderate mean connectivity, consistent with the characteristics of a scale‐free network. Therefore, β = 8 was selected for subsequent network construction (Figure 1A–C). Based on this parameter, a weighted co‐expression network and topological overlap matrix were constructed. Multiple initial modules were identified using the dynamic tree‐cutting algorithm, and highly similar modules were subsequently merged to generate the final stable module set (Figure 1D–F). Module–trait correlation analysis revealed that the MEblue module was significantly positively correlated with the BC phenotype (r = 0.79, P = 1 × 10^−18^), with overall upregulated expression in tumor samples, suggesting that it represented a candidate functional module closely associated with BC (Figure 1G). Further analysis of the relationship between module membership and gene significance within the MEblue module showed a strong positive correlation (R = 0.89, P < 1 × 10^−200^), indicating that highly representative genes within this module were also strongly associated with the BC phenotype and might possess important regulatory functions (Figure 1H).

### 3.2 EPCAM Was Identified as a Key Gene through Multi‐Algorithm Integration

**Figure 2:**
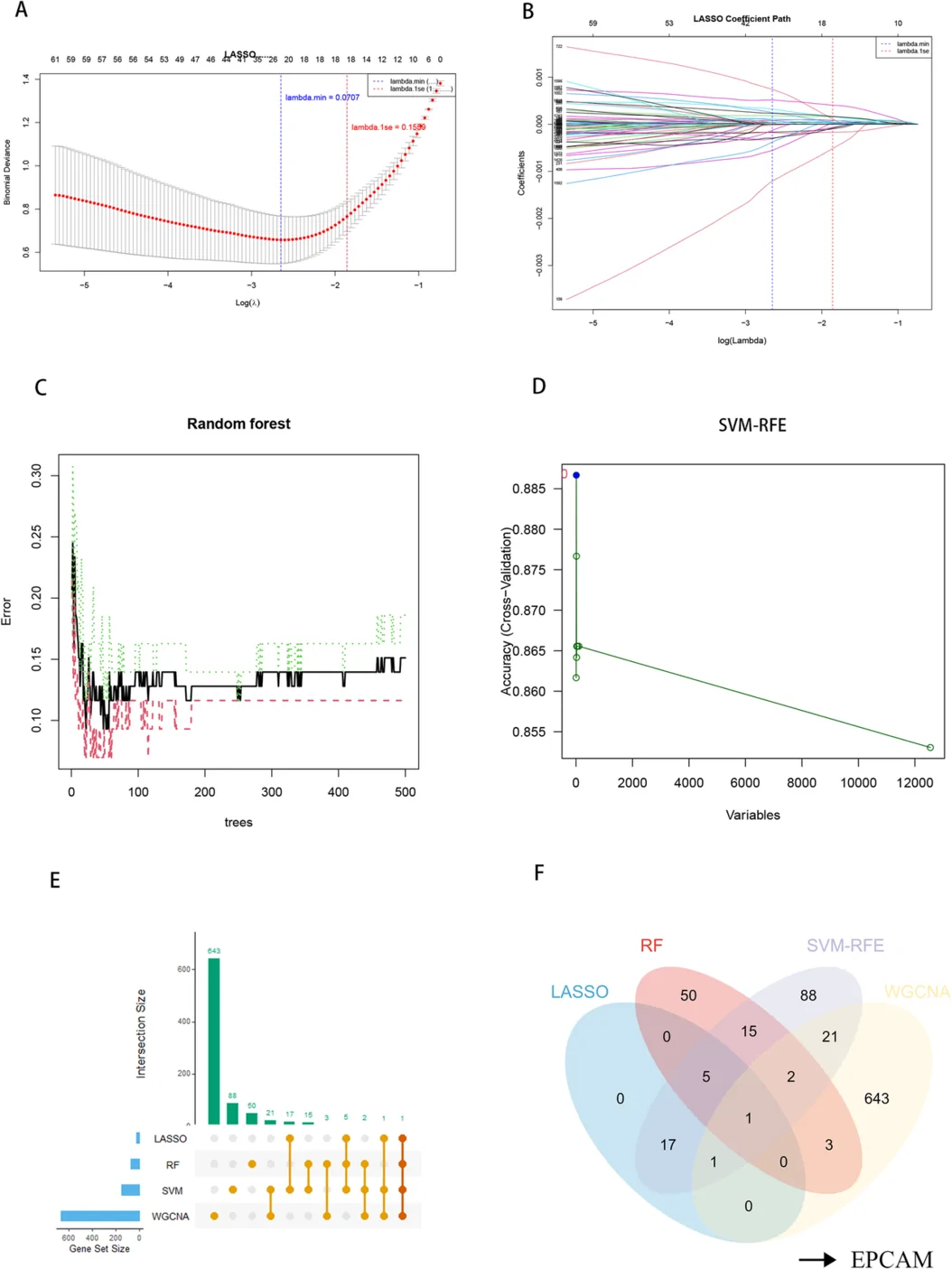
Machine learning‐based screening of differentially expressed genes and integration of key genes. A: Cross‐validation error curve of LASSO regression. Red dots represent errors under different λ values, whereas blue and red vertical lines indicate λ.min and λ.1se, respectively. B: Coefficient trajectory plot of LASSO regression showing variation trends of gene coefficients under different λ values. C: RF error curve showing gradual stabilization of model error as the number of trees increased. D: Relationship between feature number and cross‐validation accuracy in SVM‐RFE analysis, showing the optimal feature number corresponding to the highest classification accuracy. E: UpSet plot showing intersections and unions among gene sets identified by LASSO, RF, SVM‐RFE, and WGCNA. F: Venn diagram showing overlapping genes identified by the four methods, with EPCAM identified as the only common core gene.

Under 10‐fold cross‐validation, LASSO regression identified 25 feature genes using the optimal penalty parameter λ.min = 0.0707. The coefficient trajectories of these genes across different λ values also demonstrated strong stability of the selected features (Figure 2A–B). In the RF analysis, the model error gradually stabilized after the construction of 180 decision trees, and 76 key genes were identified according to gene importance scores (Figure 2C). SVM‐RFE analysis demonstrated that the highest cross‐validation accuracy was achieved when 150 variables were retained, and this variable subset was therefore selected as the optimal feature gene set (Figure 2D).

To further improve the robustness and reliability of key gene identification in BC, the outputs of the three machine learning algorithms were integrated with candidate genes identified from the MEblue module of WGCNA. UpSet and Venn diagrams were constructed to visualize the union and intersection among the four approaches. A total of 846 candidate genes were identified, among which EPCAM was the only gene shared by all four methods, indicating its high stability and biological reliability (Figure 2E,F).

### 3.3 EPCAM Was Closely Associated with Cancer‐Related and Immune‐Related Processes and Exhibited Favorable Clinical Value

**Figure 3:**
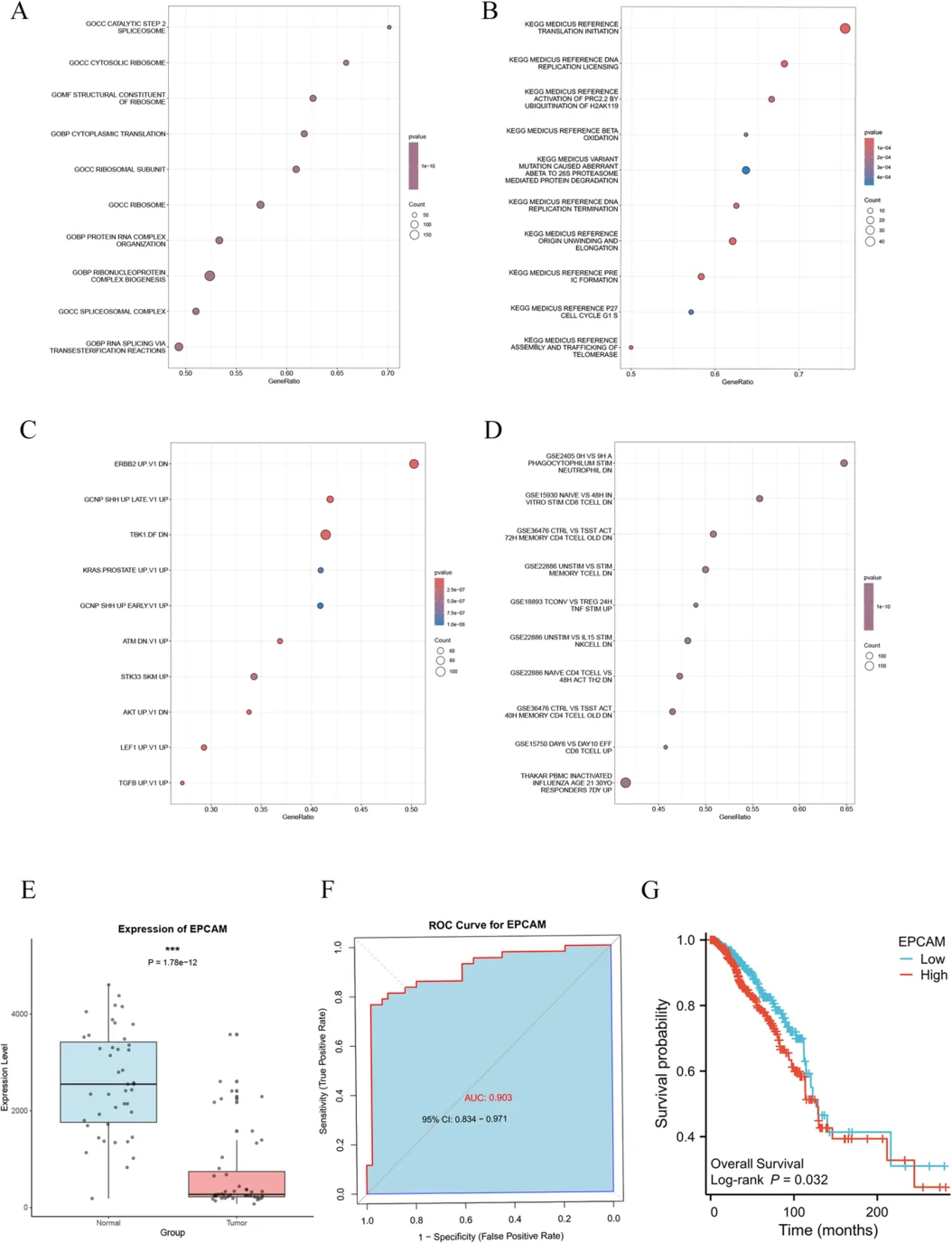
Functional enrichment and clinical value analyses of EPCAM. A: Bubble plot of GO enrichment analysis. Bubble size represents gene count, while color intensity indicates enrichment significance based on P values. B: Bubble plot and ridge plot of KEGG enrichment analysis. C: Bubble plot of oncogenic signature enrichment analysis. D: Bubble plot of immunologic signature enrichment analysis. E: Differential expression of EPCAM between BC tissues and normal tissues. F: ROC curve analysis of EPCAM. G: Kaplan–Meier survival curve analysis of EPCAM.

To systematically investigate the potential molecular mechanisms of EPCAM in BC and its role in the tumor immune microenvironment, GSEA was performed. The results demonstrated that genes positively associated with EPCAM expression were significantly enriched in multiple tumor‐related signaling pathways and immune regulatory processes (Figure 3A–D). GO enrichment analysis revealed that EPCAM was primarily involved in fundamental biological processes, including ribosomal structure, protein translation, RNA splicing, and protein complex assembly (Figure 3A). KEGG pathway analysis further showed significant enrichment in classical tumor‐related pathways, including cell cycle regulation, DNA replication, telomere maintenance, and protein degradation (Figure 3B). In addition, oncogenic signature analysis suggested that EPCAM might participate in key oncogenic signaling pathways, including ataxia telangiectasia mutated (ATM) signaling suppression, AKT pathway regulation, and transforming growth factor‐β signaling activation (Figure 3C). At the immune level, immunologic signature analysis demonstrated enrichment in immune‐related processes such as memory CD4^+^ T‐cell senescence, effector CD8^+^ T‐cell activation, and regulatory T‐cell (Treg) activity (Figure 3D). These findings suggested that EPCAM may be associated with BC progression through biological processes related to tumor cell proliferation, genomic stability, and the tumor immune microenvironment.

To further evaluate the clinical value of EPCAM in BC, its diagnostic performance and prognostic significance were validated using the independent TCGA‐BRCA cohort (Figure 3E–G). EPCAM expression was significantly elevated in BC tissues compared with normal tissues (P = 1.78 × 10^−12^) (Figure 3E), suggesting its potential involvement in tumorigenesis. ROC curve analysis yielded an AUC value of 0.903 (Figure 3F), indicating high diagnostic sensitivity and specificity. Kaplan–Meier survival analysis based on the optimal cutoff value demonstrated that patients with high EPCAM expression exhibited significantly shorter overall survival than those with low EPCAM expression (log‐rank *p*= 0.032) (Figure 3G), indicating that elevated EPCAM expression was closely associated with poor prognosis.

Collectively, these functional and clinical analyses suggested that EPCAM may be involved in tumor cell proliferation and immune‐related processes and may represent a potential diagnostic and prognostic biomarker candidate for BC.

### 3.4 Identification of Active Constituents of ZBM–TBM and Network Analysis of Their Potential Targets

**Figure 4:**
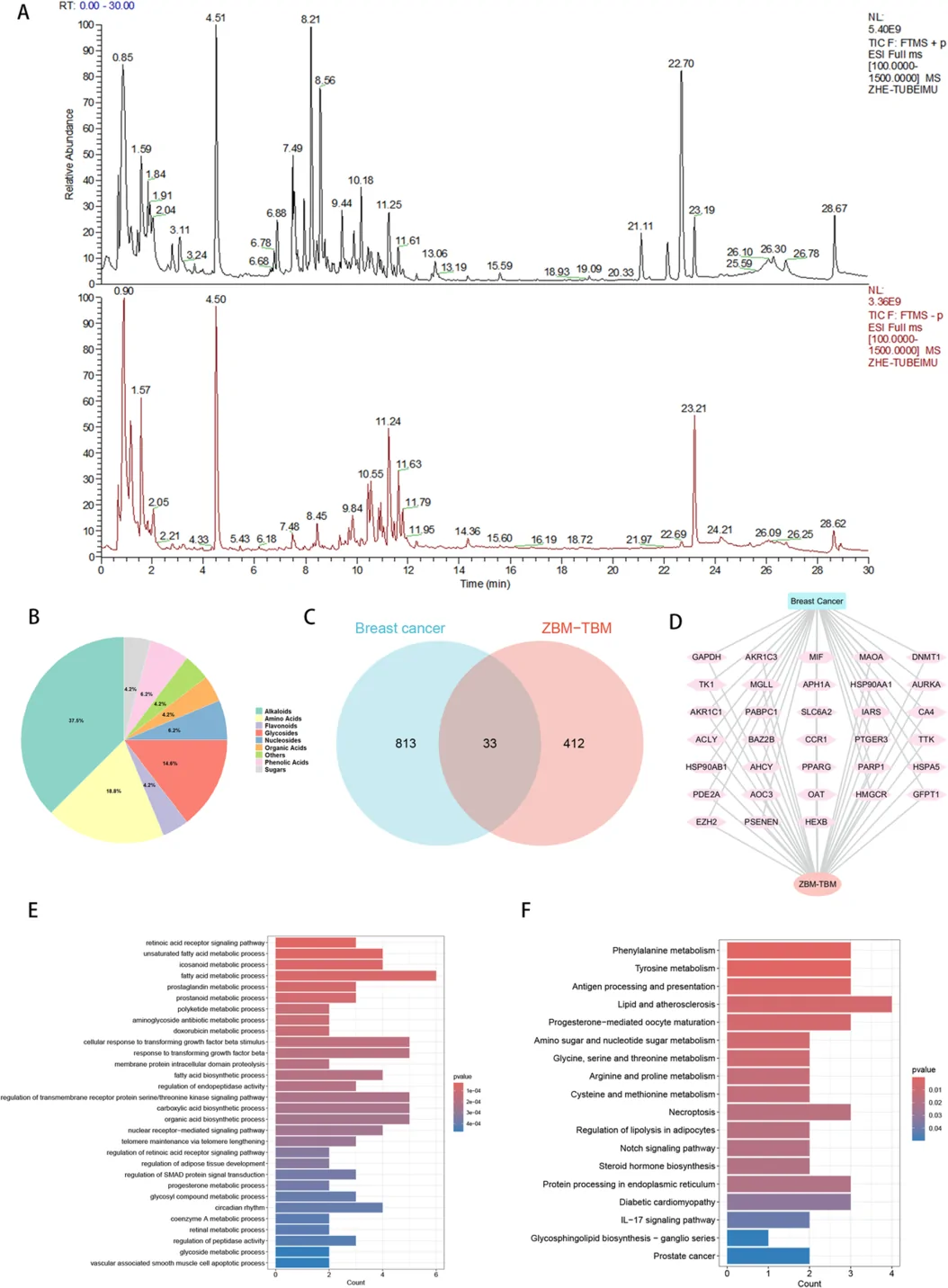
Identification of ZBM–TBM constituents, screening of overlapping targets, and functional enrichment analyses. A: Total ion chromatograms obtained by LC–MS/MS under positive ion mode (upper panel) and negative ion mode (lower panel), showing multiple chemical constituents in the samples. B: Pie chart showing the distribution of constituent categories, indicating that alkaloids were the predominant compounds, accompanied by steroids/triterpenoids, flavonoids, phenolic compounds, and organic acids. C: Venn diagram showing overlapping genes between predicted drug targets and BC‐related genes, resulting in 33 candidate genes. D: Drug–disease–target interaction network illustrating the multi‐component, multi‐target, and multi‐pathway mode of action and potential hub targets. E: Bar plot of GO enrichment analysis. F: Bar plot of KEGG pathway enrichment analysis.

LC–MS/MS analysis was performed to characterize the chemical constituents of ZBM–TBM. Clear total ion chromatograms were obtained under both positive and negative ion modes (Figure 4A), indicating the presence of multiple complex constituents in the extracts (Table 1). Major chromatographic peaks were primarily distributed within the retention time ranges of 4–10 min and 22–24 min. Further classification and statistical analysis of the identified compounds revealed that alkaloids represented the predominant constituent class, accompanied by organic acids, steroids/triterpenoids, flavonoids, phenolic compounds, and saccharides (Figure 4B). The predominance of alkaloids suggested that these compounds may represent important chemical constituents contributing to the potential pharmacological properties of ZBM–TBM, while the coexistence of multiple compound classes reflected its characteristic multi‐component synergistic effects.

**Table 1:** Hub Gene Survival Prognosis Analysis Results.

No.	Identification	t_*R*_(min)	Formula	Observed mass (Da)	Calculated mass (Da)	Mass error (ppm)	Adducts	Ms/Ms(m/*z*)	
1	DL‐Arginine	0.76	C6H14N4O2	175.1186	175.1190	−2.28	[M + H]+		175.1186, 158.0921, 116.0704
2	Ornithine	0.81	C5H12N2O2	131.0825	131.0826	−0.76	[M‐H]‐		131.0825, 114.0197, 70.0298
3	l‐citrulline	0.85	C6H13N3O3	176.1027	176.1030	−1.70	[M + H]+		176.1027, 159.0761, 113.0707, 70.0650
4	Sucrose	0.86	C12H22O11	341.1094	341.1089	1.47	[M‐H]‐		341.1094, 179.0569, 161.0459, 119.0353, 101.0247, 89.0247, 71.0141, 59.0140
5	Valine	0.99	C5H11NO2	118.0860	118.0863	−2.54	[M + H]+		118.0860, 72.0806, 59.0739,
6	α, α‐Trehalose	1.04	C12H22O11	341.1085	341.1089	−1.17	[M‐H]‐		341.1058, 179.0560, 161.0455, 119.0349, 101.0243, 71.0138
7	L‐(‐)‐Malic acid	1.09	C4H6O5	133.0141	133.0142	−0.75	[M‐H]‐		133.0141, 115.0036, 89.0243, 72.9931
8	Citric acid	1.19	C6H8O7	191.0197	191.0197	0.00	[M‐H]‐		191.0197, 111.0087, 87.0087, 85.0295
9	Pyroglutamic acid	1.37	C5H7NO3	130.0496	130.0498	−1.54	[M + H]+		130.0496, 84.0442
10	l‐homoproline	1.43	C6H11NO2	130.0863	130.0860	2.31	[M + H]+		130.0863, 70.0650,
11	Nicotinic acid	1.53	C6H5NO2	124.0390	124.0393	−2.42	[M + H]+		124.0390, 78.0337
12	4‐hydroxyproline	1.56	C7H11NO4	174.0758	174.0761	−1.72	[M + H]+		174.0758, 128.0704, 110.0598
13	Uridine	1.68	C9H12N2O6	243.0621	243.0623	−0.82	[M‐H]‐		243.0621, 153.0305, 152.0352, 110.0247
14	Uracil	1.69	C4H4N2O2	113.0343	113.0345	−1.77	[M + H]+		113.0343, 70.0286, 42.0337
15	M‐Coumaric acid	1.75	C9H8O3	165.0543	165.0546	−1.82	[M + H]+		165.0543, 91.0540, 95.0489, 119.0489, 123.0438, 147.0438
16	l‐Tyrosine	1.75	C9H11NO3	182.0808	182.0812	−2.20	[M + H]+		182.0808, 165.0543, 136.0754, 123.0438, 91.0540
17	Isoleucine	1.91	C6H13NO2	132.1016	132.1019	−2.27	[M + H]+		132.1019, 86.0962
18	Adenosine	1.98	C10H13N5O4	268.1036	268.1040	−1.49	[M + H]+		268.1036, 136.0615, 57.0334
19	5‐Hydroxymethylfurfural	2.27	C6H6O3	127.0387	127.0390	−2.36	[M + H]+		127.0387, 109.0282, 81.0333, 71.0490, 69.0333, 53.0384
20	Peimisine N‐oxide	6.88	C27H42O4N	444.3100	444.3108	−1.80	[M + H]+		444.31000, 409.2735, 353.2107
21	Vanilpyruvic acid	7.20	C10 H10 O5	209.0455	209.0455	0.00	[M‐H]‐		209.0455, 165.0555, 121.0295,
22	Rutin	7.25	C27H30O16	609.1460	609.1461	−0.16	[M‐H]‐		609.1460, 300.0274, 301.0352
23	Quercetin‐3‐o‐rutinose	7.32	C27H30O16	609.1460	609.1461	−0.16	[M‐H]‐		609.1460, 300.0275, 301.0352
24	1‐O‐Glycerol ferulate	7.35	C13H16O6	267.0872	267.0874	−0.75	[M‐H]‐		267.0872, 175.0400, 252.0638, 133.0294, 149.0607
25	Sipeimine glycoside	7.57	C33H53NO8	592.3841	592.3843	−0.34	[M + H]+		574.3726、480.5835、412.3194
26	Peimisine	7.92	C27H41NO3	428.3149	428.3159	−2.33	[M + H]+		428.3149, 67.0540, 81.0697, 84.0806, 393.2783
27	Peimine	8.08	C27H45NO3	432.3469	432.3472	−0.69	[M + H]+		432.3469, 414.3373, 98.0962
28	Peiminine	8.08	C27H43NO4	446.3258	446.3264	−1.34	[M + H]+		446.3258, 98.0963, 105.0698
29	Peimine	8.21	C27H45NO3	432.3463	432.3472	−2.08	[M + H]+		432.3463, 414.3358
30	Isomer of zhebeininoside	8.22	C33H55NO8	594.3988	594.4000	−2.02	[M + H]+		594.3988, 576.3881, 98.0962, 112.1118, 414.3366
31	Zhebeininoside	8.28	C33H56O8N	594.3987	594.4000	−2.19	[M + H]+		594.3987, 576.3882
32	Zhebeinone	8.56	C27H43NO3	430.3306	430.3315	−2.09	[M + H]+		430.3306, 412.3202
33	Sipeimine glycoside	8.76	C33H53NO8	592.3831	592.3843	−2.03	[M + H]+		592.3831, 412.3203, 98.0963,
34	Isopeimisine	8.89	C27H41NO3	428.3151	428.3159	−1.87	[M + H]+		428.3151, 67.0540, 81.0697, 84.0806
35	Yibeinoside A	9.18	C33H53NO7	576.3881	576.3895	−2.43	[M + H]+		576.3881, 414.3358, 98.0962, 85.0282, 396.3246
36	Isopeimine	9.44	C27H45NO3	432.3461	432.3472	−2.54	[M + H]+		432.3461, 414.3357
37	Neopetasin	9.53	C27H43NO2	414.3358	414.3366	−1.93	[M + H]+		414.3358, 98.0963,
38	Hubeibeisine glycoside	9.61	C33H55NO7	578.4038	578.4051	−2.25	[M + H]+		578.4038, 416.3516,
39	Pingbeimine	9.61	C33H55NO7	578.4038	578.4051	−2.25	[M + H]+		578.4038, 416.3515
40	Zhebeirine	9.80	C27H43NO2	414.3358	414.3366	−1.93	[M + H]+		414.3357, 396.3252, 98.0962
41	Peiminine	10.15	C27H43NO4	446.3255	446.3264	−2.02	[M + H]+		446.3255, 98.0963, 105.0697
42	Sipeimine	10.19	C27H43NO3	430.3307	430.3315	−1.86	[M + H]+		430.3306, 412.3207
43	Ebeiedinone	10.38	C27H43NO2	414.3358	414.3366	−1.93	[M + H]+		414.3357, 396.3248, 98.0962
44	Peiminine	10.54	C27H43NO4	446.3255	446.3264	−2.02	[M + H]+		446.3255, 98.0963, 105.0697
45	Isomer of ebeiedinone	10.74	C27H43NO2	414.3356	414.3366	−2.41	[M + H]+		414.3356, 98.0962, 93.0697, 67.0540, 81.0697, 119.0851, 105.0697
46	Puqiedinone	10.80	C27H43NO2	414.3356	414.3366	−2.41	[M + H]+		414.3356, 98.0962, 93.0697, 67.0539, 81.0697, 119.0853, 105.0697, 396.3244
47	Ebeiedine	10.96	C27H45NO2	416.3513	416.3523	−2.40	[M + H]+		416.3513, 98.0962, 79.0554, 67.0549
48	Puqiedine	11.03	C27H45NO2	416.3515	416.3523	−1.92	[M + H]+		416.3515, 55.0542, 98.0961, 81.0697

After further screening and removal of irrelevant variables, 32 key active constituents of ZBM–TBM were retained. Potential target prediction of these compounds followed by intersection analysis with BC‐related genes identified 33 candidate overlapping genes (Figure 4C). Subsequently, a drug–disease–target interaction network was constructed (Figure 4D), revealing complex relationships between active constituents and multiple targets and indicating a potential multi‐component, multi‐target, and multi‐pathway regulatory pattern. Several targets exhibited relatively high connectivity within the network, suggesting their potential involvement in the biological effects of ZBM–TBM against BC.

To further investigate the biological functions of the candidate genes, GO and KEGG enrichment analyses were performed. GO enrichment analysis demonstrated that these genes were mainly involved in metabolic processes related to lipids and amino acids, receptor and transcriptional regulation, apoptosis, and stress responses (Figure 4E). At the cellular component and molecular function levels, these genes were enriched in membrane receptor complexes, cytoplasmic and nuclear localization, and were mainly associated with enzymatic activity, receptor activity, and binding functions. KEGG enrichment analysis further revealed that these genes were significantly involved in amino acid and glycolipid metabolism pathways, as well as tumor‐related signaling pathways including Notch and interleukin‐17 signaling pathways, together with steroid hormone biosynthesis and protein processing in the endoplasmic reticulum (Figure 4F). Several enriched pathways were also associated with cardiovascular diseases, metabolic disorders, and cancers, suggesting that ZBM–TBM may exert antitumor effects through integrated mechanisms involving metabolic reprogramming and immune/inflammatory signaling regulation.

### 3.5 Most Hub Genes Exhibited Favorable Prognostic Value

**Figure 5:**
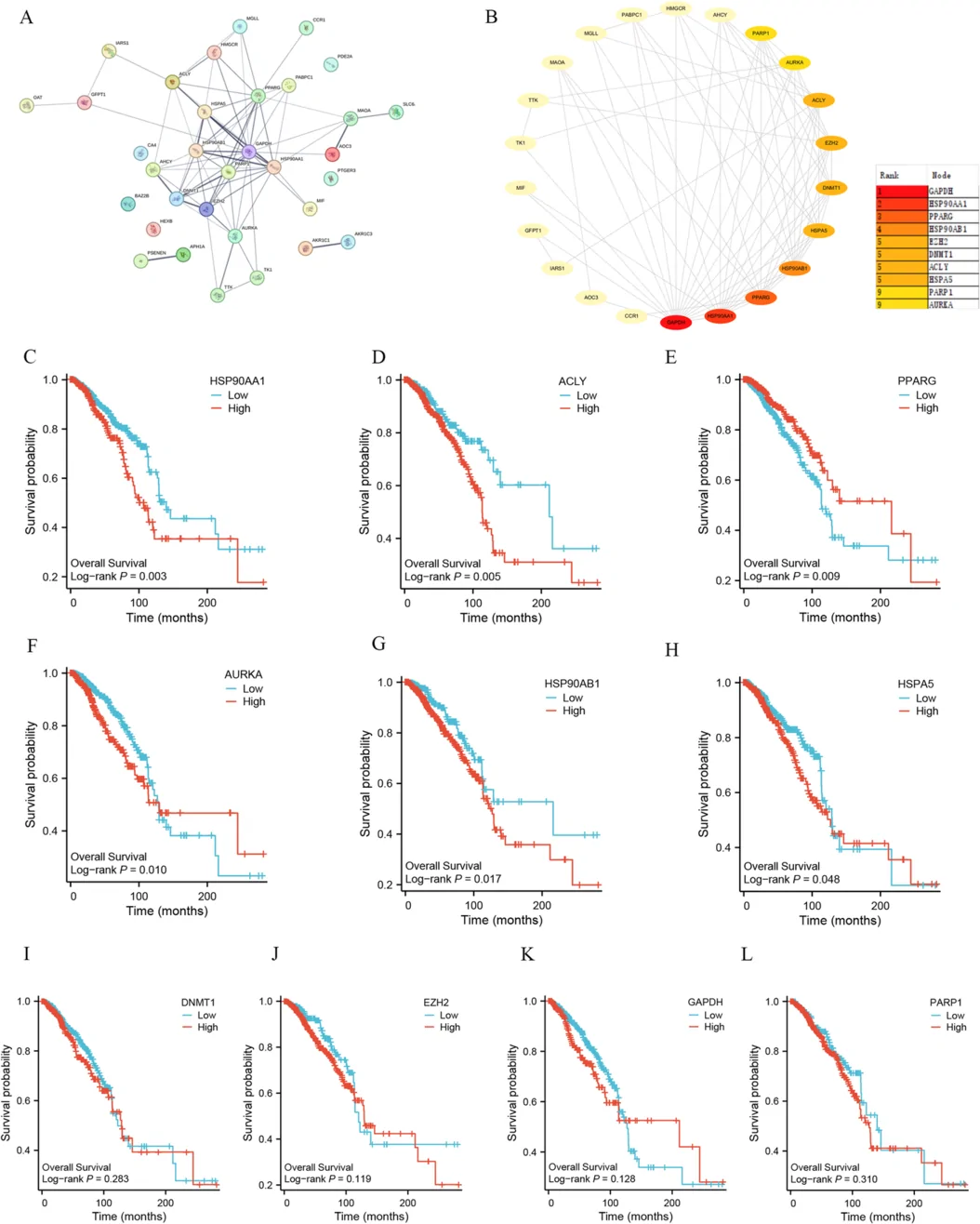
Identification of hub genes and survival analyses. A: PPI network of overlapping genes. Nodes represent genes, whereas edges indicate interactions between genes. Color intensity reflects node connectivity. B: Identification of hub genes and MCC score ranking among overlapping genes. Colors ranging from red to yellow indicate different MCC scores. C–H: Kaplan–Meier survival curves of hub genes with significant prognostic value (*p* < 0.05). I–L: Kaplan–Meier survival curves of hub genes without significant prognostic association.

To further identify core genes with key regulatory functions among the overlapping genes, PPI network analysis combined with node centrality analysis using the cytoHubba plugin was performed. The top 10 hub genes ranked by MCC scores were identified as heat shock protein 90 alpha family class A member 1 HSP90AA1, ACLY, PPARG, AURKA, HSP90AB1, HSPA5, EZH2, GAPDH, DNMT1, and PARP1 (Figure 5A,B). These genes may be involved in biological processes associated with BC development and progression.

To evaluate the clinical prognostic significance of these hub genes, survival analyses were conducted using the TCGA‐BRCA cohort. Kaplan–Meier survival analysis demonstrated that high expression levels of six hub genes were significantly associated with poorer overall survival in patients with BC (log‐rank *p* < 0.05) (Figure 5C–L). These findings suggested that the identified hub genes may serve as potential prognostic biomarkers for BC and that their elevated expression may be associated with malignant characteristics.

### 3.6 Immune Cell Landscape of BC and Immune Correlation Analysis of Hub Genes

**Figure 6:**
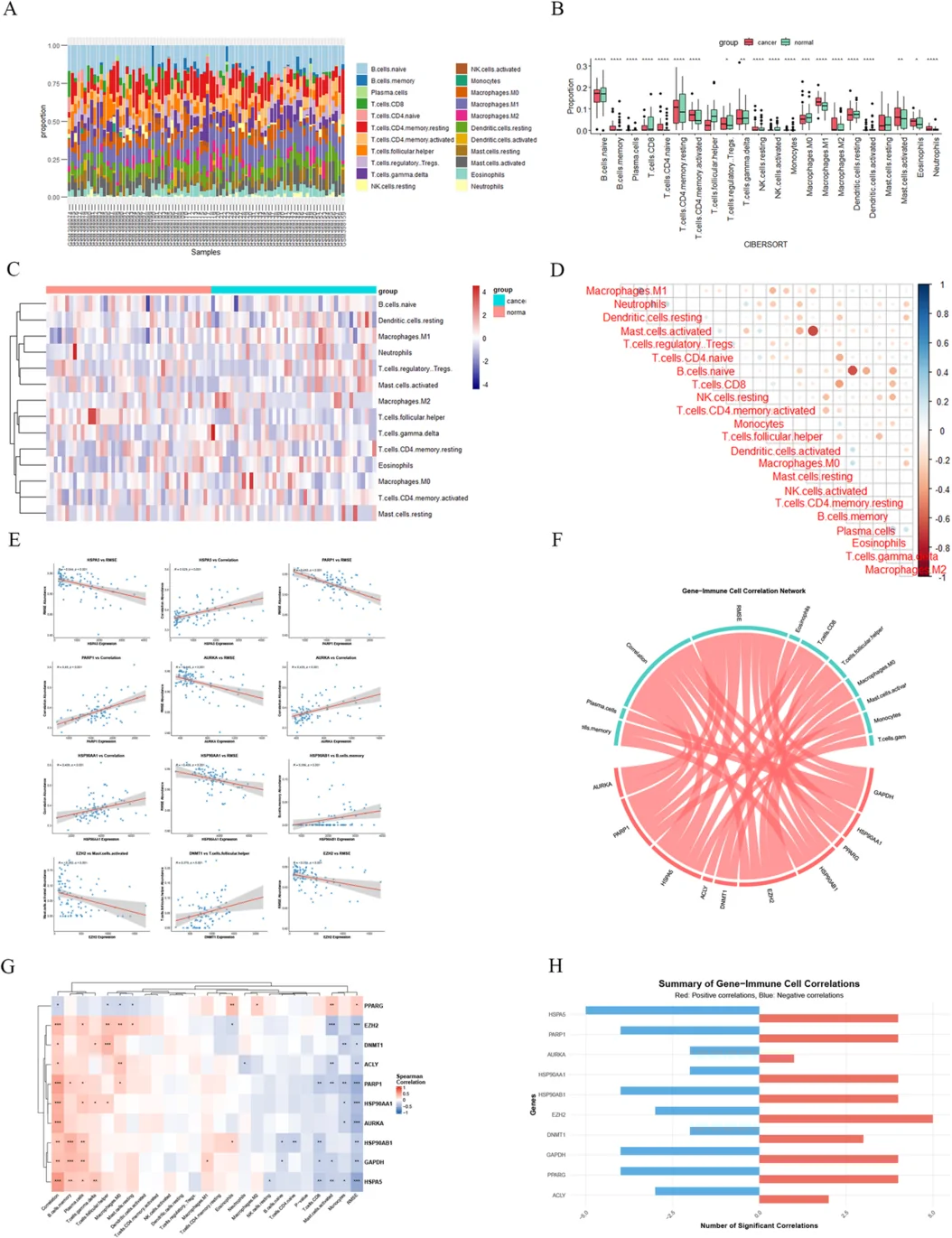
Immune cell landscape of BC tissues and immune correlation analyses of hub genes. A: Stacked bar plot of immune cell proportions. Different colors represent different immune cell types, reflecting their relative proportions in each sample. B: Differential analysis of immune cell infiltration estimated by CIBERSORT. C: Heatmap of immune cell distributions across different samples. Red indicates high abundance, whereas blue indicates low abundance. D: Correlation plot of immune cell subtypes. Red indicates positive correlations, whereas blue indicates negative correlations. E: Scatter plots showing correlations between hub genes and immune cell subtypes. Red lines represent regression curves, gray shaded areas indicate 95% confidence intervals, and P values represent statistical significance. F: Correlation network between hub genes and immune cell subtypes. Chord diagrams illustrate positive and negative correlations. G: Heatmap of Spearman correlations between hub genes and immune infiltration levels. Red indicates positive correlations, whereas blue indicates negative correlations. Color intensity reflects correlation strength, and asterisks indicate significance levels. H: Bar plot summarizing the number of significant correlations between each hub gene and immune cell subtypes.

To systematically characterize alterations in the BC immune microenvironment and explore their potential molecular regulatory mechanisms, comprehensive analyses integrating CIBERSORT‐based immune infiltration analysis and hub gene immune correlation analysis were performed (Figure 6).

Immune infiltration analysis revealed significant differences in immune cell composition between BC tissues and normal tissues (Figure 6A,B). Overall, the proportions of immune effector cells, including B cells and CD4^+^ memory T cells, were markedly decreased in tumor tissues, whereas immunosuppressive cells such as Treg cells were relatively increased, indicating a typical immunosuppressive microenvironment in BC tissues. Heatmap analysis further demonstrated that macrophage M1 cells and activated CD4^+^ memory T cells exhibited relatively high abundance in tumor samples compared with normal tissues (Figure 6C), suggesting functional remodeling of immune cells within the tumor microenvironment. Moreover, complex correlation networks were observed among different immune cell subtypes. For example, macrophage M1 cells were negatively correlated with Treg cells, whereas CD8^+^ T cells showed positive correlations with helper T cells (Figure 6D), further reflecting the dynamic regulatory characteristics of the BC immune microenvironment.

To further elucidate the molecular basis underlying the observed immune infiltration patterns, correlations between hub genes and immune cell infiltration were subsequently analyzed (Figure 6E–H). The results demonstrated that most hub genes exhibited significant correlations with multiple immune cell subtypes. Specifically, PARP1, HSP90AA1, and HSP90AB1 were positively correlated with B cells, follicular helper T (Tfh) cells, and M0 macrophages, whereas HSPA5, EZH2, and AURKA showed negative correlations with mast cells and monocytes, suggesting distinct immunoregulatory roles of different hub genes. Furthermore, gene–immune cell interaction network analysis revealed that HSPA5, PARP1, and EZH2 possessed relatively high connectivity and exhibited typical hub characteristics, being broadly associated with multiple immune cell populations (Figure 6G). These findings suggested that these genes may be associated with immune microenvironmental characteristics in BC.

### 3.7 Computational Prediction of Potential Regulatory Associations between EPCAM and Hub Genes

Previous analyses identified EPCAM as a candidate key gene associated with BC and revealed its potential involvement in tumor‐related and immune‐related biological processes. However, EPCAM was not identified as a direct predicted target of the ZBM–TBM herb pair based on the drug–target prediction analysis. Therefore, further computational analyses were performed to investigate the potential associations between EPCAM and hub genes identified from the ZBM–TBM‐related regulatory network.

**Figure 7:**
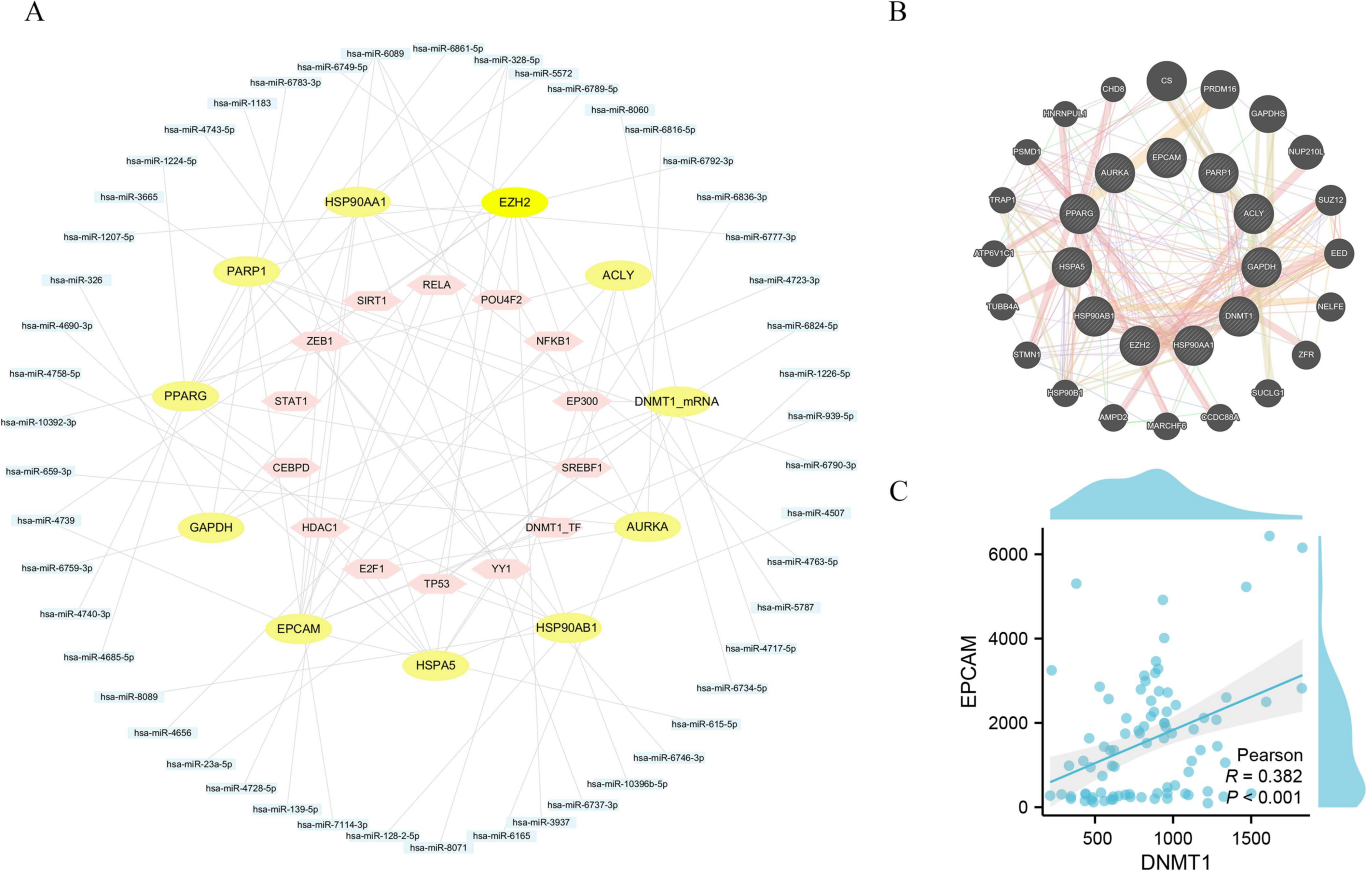
TF–mRNA–miRNA regulatory network, gene co‐occurrence network, and correlation analysis between EPCAM and DNMT1. A: Computational TF–mRNA–miRNA association network constructed based on TRRUST and miRWalk databases. B: Gene co‐occurrence network constructed using the GeneMANIA platform. C: Scatter plot showing the expression correlation between EPCAM and DNMT1.

A TF–mRNA–miRNA regulatory network was constructed by integrating experimentally reported TF–target interactions and predicted miRNA–mRNA interactions (Figure 7A). The network revealed complex regulatory relationships involving multiple hub genes, including DNMT1, ACLY, PARP1, HSP90AA1, PPARG, EZH2, HSPA5, and EPCAM. Among these genes, DNMT1 showed potential associations with EPCAM within the integrated network, suggesting that DNMT1 may be involved in EPCAM‐associated biological processes. However, the TF–mRNA–miRNA network alone cannot determine definitive upstream or downstream regulatory relationships, and these predicted interactions require further experimental validation.

To further explore the functional associations among EPCAM and hub genes, a gene co‐occurrence network was constructed using GeneMANIA (Figure 7B). EPCAM exhibited functional associations with multiple hub genes, including DNMT1 and AURKA, based on co‐expression, physical interaction, pathway association, and related functional annotations. These findings suggested that EPCAM may be connected with ZBM–TBM‐associated candidate targets through a broader molecular interaction network rather than through a single linear regulatory pathway.

Furthermore, gene expression correlation analysis demonstrated a significant positive correlation between EPCAM and DNMT1 expression levels (R = 0.38, *p* = 0.00029) (Figure 7C). This correlation indicated a coordinated expression pattern between EPCAM and DNMT1 in BC samples. Considering the known involvement of DNMT1 in epigenetic regulation, these findings suggested that DNMT1 may be associated with EPCAM‐related biological processes. Nevertheless, correlation analysis and computational network prediction cannot establish direct regulatory causality.

Collectively, these analyses suggest that EPCAM may be indirectly associated with the ZBM–TBM‐related molecular network through multiple candidate hub genes, including DNMT1 and AURKA. The proposed relationships among active compounds, candidate targets, and EPCAM‐associated biological processes represent a computationally predicted regulatory framework rather than a confirmed molecular axis. Further in vitro and in vivo experiments are required to clarify the upstream and downstream relationships among ZBM–TBM, peiminine, hub genes, and EPCAM.

### 3.8 Molecular Docking and Visualization

**Figure 8:**
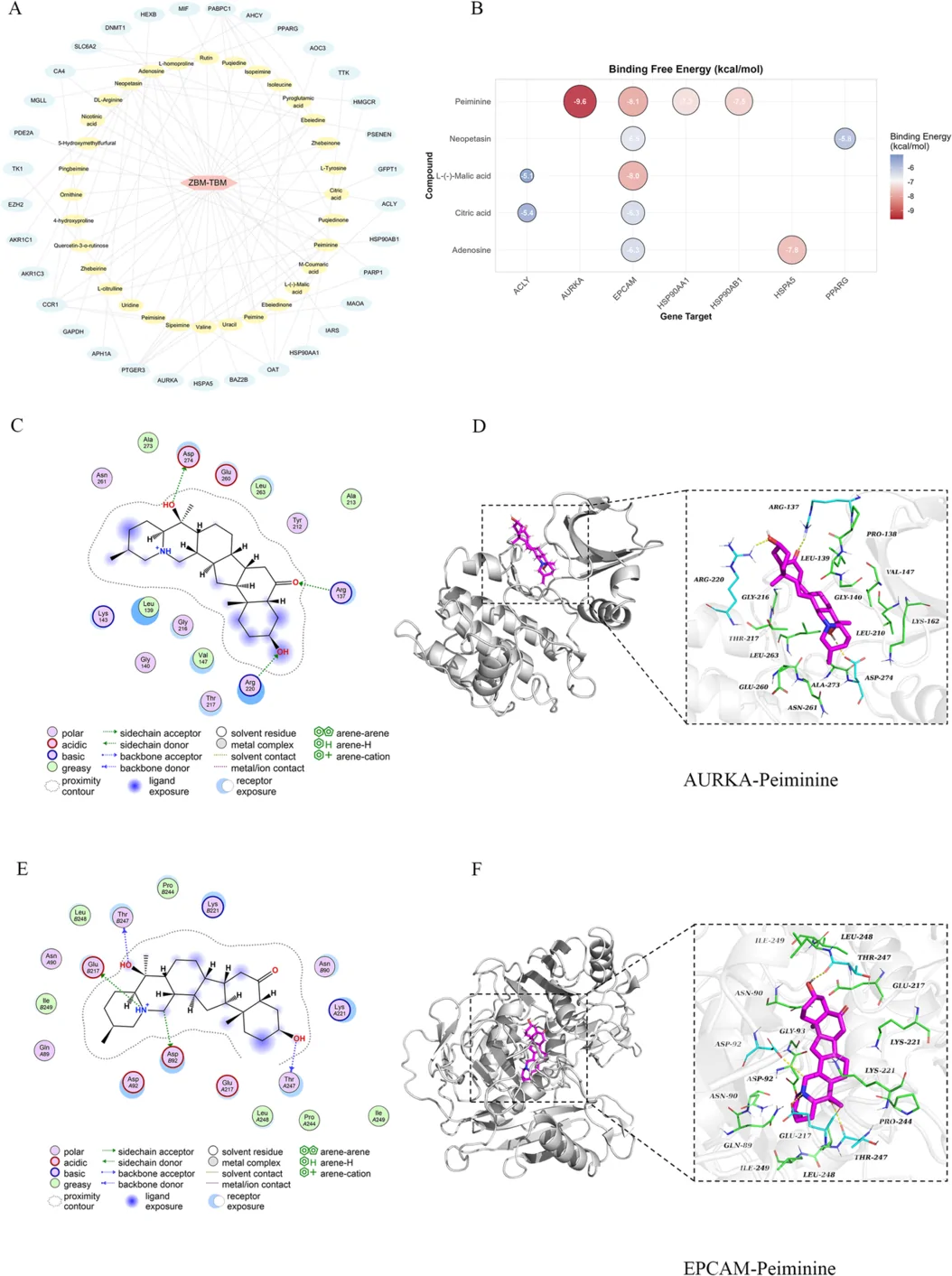
Molecular docking and visualization analyses. A: Drug–compound–target interaction network showing interactions between active constituents and potential target proteins. B: Bubble plot of binding free energies between active constituents and target proteins. Red indicates lower binding free energy and stronger binding affinity. C, E: Two‐dimensional interaction diagrams showing non‐covalent interactions between peiminine and AURKA/EPCAM, including hydrogen bonds, hydrophobic interactions, and π‐related interactions. D, F: Three‐dimensional binding conformations of peiminine with AURKA and EPCAM, highlighting binding pockets and surrounding key residues.

To further investigate the potential interactions between active constituents of ZBM–TBM and key targets at the molecular level, a drug–compound–target interaction network was constructed, followed by molecular docking analysis to evaluate the core interaction relationships (Figure 8A,B). Network analysis demonstrated that most active constituents could simultaneously interact with multiple targets, while several hub genes exhibited high connectivity within the network, suggesting their critical roles in the therapeutic effects of ZBM–TBM against BC. Based on these findings, core prognostically significant targets and their corresponding active constituents were selected for molecular docking analysis.

Notably, although previous analyses indicated that EPCAM was not a direct target of ZBM–TBM, it remained an important phenotype‐associated molecule in BC and exhibited associations with hub genes. Therefore, supporting molecular docking analysis and subsequent MD simulations involving EPCAM were further conducted to explore the potential molecular interaction characteristics between EPCAM and active constituents. The docking results suggested that several active constituents exhibited strong binding capacities toward key targets, among which peiminine showed binding free energies below −7.0 kcal/mol with multiple hub genes (Figure 8B), suggesting favorable predicted binding characteristics and indicating that peiminine may represent a potential key active constituent.

Further two‐dimensional and three‐dimensional structural analyses were performed for the representative complexes AURKA–peiminine and EPCAM–peiminine, which exhibited the lowest binding free energies (Figure 8C–F). Two‐dimensional interaction analysis revealed that peiminine formed multiple types of non‐covalent interactions with surrounding amino acid residues during binding, including hydrogen bonds, hydrophobic interactions, and π‐related interactions (Figure 8C,E). These interactions were mainly distributed around key residues surrounding the ligand and contributed to maintaining a stable ligand conformation within the binding site. Three‐dimensional structural analysis further demonstrated that peiminine could be deeply embedded within the binding pockets of the target proteins and form extensive spatial interaction networks with surrounding residues (Figure 8D,F). In the AURKA complex, the ligand occupied the typical kinase domain binding region and established tight spatial interactions with adjacent residues. Similarly, in the EPCAM complex, peiminine showed a predicted binding pose within a potential binding region and exhibited favorable computationally predicted spatial interactions.

Collectively, both two‐dimensional and three‐dimensional structural analyses demonstrated that peiminine formed relatively stable and favorable binding conformations with AURKA and EPCAM. These findings provide computational support for AURKA as a potential therapeutic target candidate and provide computational evidence suggesting a possible interaction between peiminine and EPCAM.

### 3.9 Molecular Dynamics Simulations

**Figure 9:**
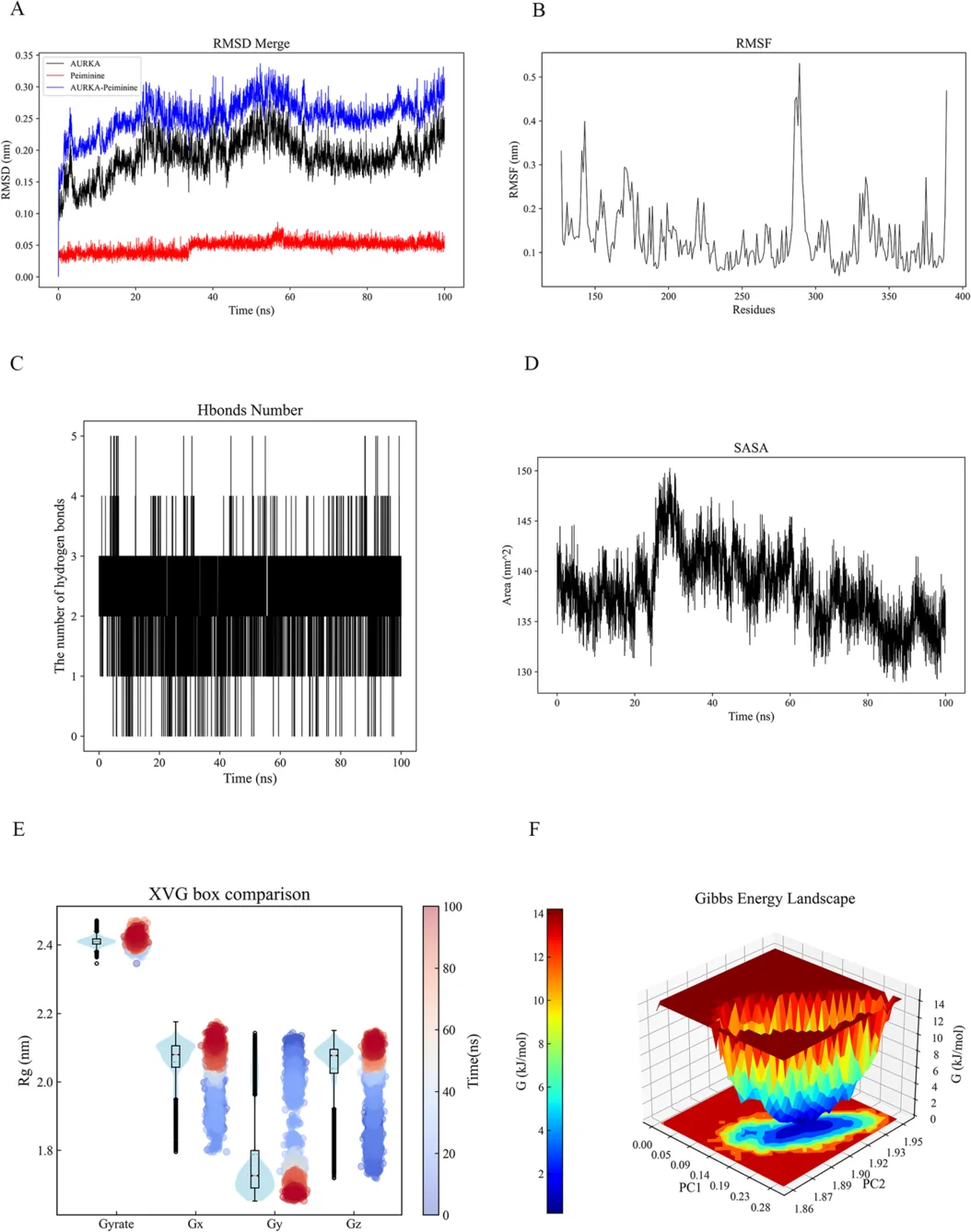
MD simulations of the AURKA–peiminine and EPCAM–peiminine complexes. A: RMSD profiles of proteins, ligands, and complexes throughout the simulation period. B: RMSF analysis showing residue‐level flexibility of proteins. C: Number of hydrogen bonds formed between proteins and ligands during the simulation. D: Variation in solvent‐accessible surface area over time. E: Rg analysis indicating structural compactness during the simulation. F: Gibbs free energy landscapes showing conformational stability of the complexes.

In the MD simulations, two protein–ligand systems, AURKA–peiminine and EPCAM–peiminine, were each subjected to 100 ns simulations to evaluate their structural stability and interaction characteristics in a solvent environment (Figure 9 and Supporting Figure 1).

For the AURKA–peiminine system, RMSD analysis demonstrated that the complex rapidly reached equilibrium during the initial stage of the simulation and remained relatively stable throughout the simulation period (Figure 9A). The RMSD fluctuations of the protein and the entire complex were relatively small, with average values of 0.19 ± 0.03 nm and 0.25 ± 0.03 nm, respectively, indicating overall structural stability of the system. The ligand RMSD exhibited an average value of 0.05 ± 0.01 nm, suggesting that peiminine remained stably positioned within the binding pocket without obvious displacement. RMSF analysis further revealed the flexibility distribution of individual residues within the protein (Figure 9B). Overall, most residues displayed low fluctuation amplitudes, with only a few local regions exhibiting relatively higher flexibility, indicating that the protein structure remained globally stable while retaining limited local flexibility. Hydrogen bond analysis demonstrated the formation of stable hydrogen bond interactions between the protein and ligand throughout the simulation process (Figure 9C). The number of hydrogen bonds was primarily distributed between one and three, with an average of approximately two hydrogen bonds, suggesting sustained intermolecular interactions within the complex. Solvent‐accessible surface area analysis indicated minimal fluctuation in solvent exposure during the simulation (Figure 9D), with an average value of 138.17 ± 3.51 nm^2^, suggesting that no obvious conformational unfolding occurred and that the complex maintained a relatively stable solvent‐exposed state. Analysis of Rg further demonstrated that the structural compactness of the protein remained stable during the simulation (Figure 9E), further supporting the overall conformational stability of the system.

In addition, free energy landscape analysis revealed relatively concentrated low‐energy regions within the system (Figure 9F), suggesting that the complex predominantly maintained stable conformational states during the simulation. According to MM/GBSA calculations, the total binding free energy was −36.03 ± 1.01 kcal/mol, in which van der Waals interactions (ΔVDWAALS =−47.08 ± 0.69 kcal/mol) and electrostatic interactions (ΔEelec = −15.21 ± 0.63 kcal/mol) served as the major driving forces, whereas polar solvation energy (ΔEGB = 31.96 ± 0.38 kcal/mol) partially offset the binding contribution. Overall, the negative binding free energy indicated strong binding affinity between peiminine and AURKA. The EPCAM–peiminine system also demonstrated relatively favorable simulation characteristics, although slightly weaker than those observed for the AURKA–peiminine system. These findings suggested that peiminine may have potential molecular interaction characteristics with EPCAM under computational simulation conditions; however, whether EPCAM represents a direct biological target requires further experimental validation.

Taken together, the simulation results suggested that both the AURKA–peiminine and EPCAM–peiminine systems maintained relatively stable conformations and favorable predicted binding characteristics during the simulation period. Structural parameters remained within relatively stable ranges, hydrogen bond interactions were persistent throughout the simulation, and MM/GBSA binding free energies remained significantly negative. These findings indicated that peiminine could effectively occupy the binding pockets of target proteins and maintain dynamic equilibrium of the complexes, thereby providing computational evidence supporting its potential regulatory effects on relevant molecular pathways.

### 3.10 Single‐Cell Virtual Knockout Analysis

**Figure 10:**
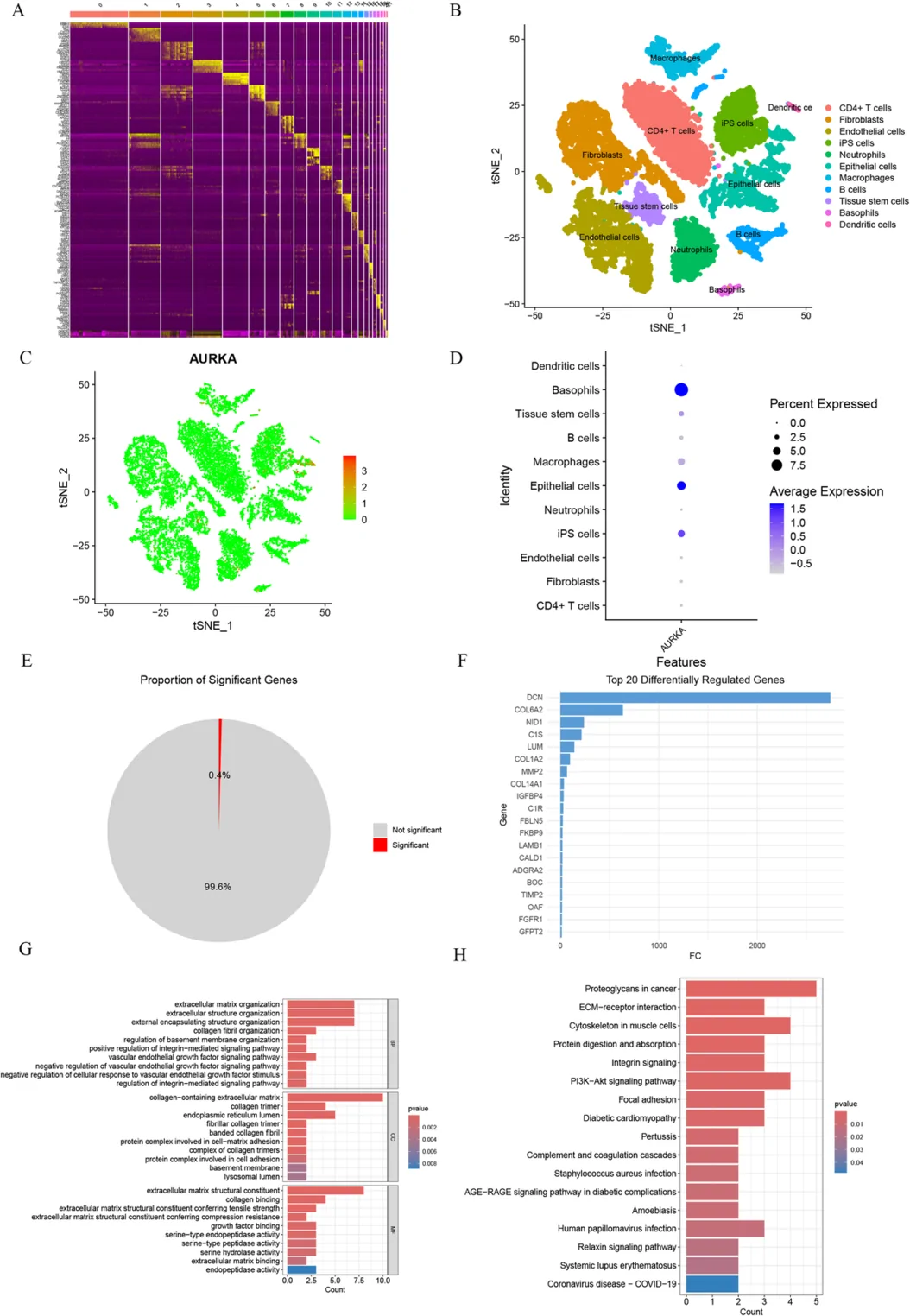
Single‐cell virtual knockout analysis. A: Heatmap of differentially expressed genes showing gene expression patterns and stratification characteristics among different cell clusters. B: t‐SNE clustering plot showing the distribution of multiple characteristic cell subpopulations with corresponding cell‐type annotations. C: Distribution map of AURKA expression at the single‐cell level, illustrating its spatial expression patterns across different cell clusters. D: Bubble plot of AURKA expression in different cell types. Bubble size represents the proportion of expressing cells, and color intensity indicates the average expression level. E: Statistical plot showing the proportion of significantly differentially expressed genes after virtual knockout, reflecting the overall transcriptomic impact of perturbation. F: Ranked bar plot of differentially expressed genes showing representative genes with marked expression alterations.

To investigate the potential functional relevance of AURKA at the cellular level, single‐cell virtual knockout analysis was performed based on single‐cell RNA sequencing data, followed by systematic investigation of cell distribution characteristics, gene expression alterations, and functional pathways (Figure 10).

The heatmap of differentially expressed genes revealed distinct expression stratification among different cell clusters, with each cluster exhibiting relatively independent gene expression patterns (Figure 10A), indicating marked cellular heterogeneity within BC tissues. t‐SNE dimensionality reduction further classified cells into multiple representative subpopulations, including CD4^+^T cells, fibroblasts, endothelial cells, epithelial cells, neutrophils, macrophages, and B cells (Figure 10B), with clear separation among clusters. Expression analysis demonstrated that AURKA exhibited low‐to‐moderate overall expression across cell populations but showed relatively elevated expression in specific cell subsets (Figure 10C,D), suggesting potential functional roles in particular cellular states.

Differential expression analysis following computational virtual knockout suggested that approximately 0.4% of genes exhibited significant expression alterations (Figure 10E), indicating that computational perturbation of AURKA expression was associated with relatively specific transcriptomic changes. Ranking analysis of differentially expressed genes further revealed that the major altered genes were primarily associated with extracellular matrix‐related molecules, including decorin (DCN), collagen type VI alpha 2 chain (COL6A2), and collagen type I alpha 2 chain (COL1A2) (Figure 10F).

Functional enrichment analysis indicated that these differentially expressed genes were mainly enriched in cell cycle‐related pathways at the GO level, including DNA replication, mitosis, and chromosome segregation, as well as extracellular matrix organization, collagen fibril formation, cell adhesion, and angiogenesis‐related processes (Figure 10G). KEGG pathway analysis further demonstrated significant enrichment in tumor‐related pathways, including extracellular matrix–receptor interaction, phosphoinositide 3‐kinase/protein kinase B signaling pathway, and focal adhesion (Figure 10H). Collectively, these findings suggested that AURKA‐associated cell cycle and cell adhesion pathways may be related to EPCAM‐associated biological processes.

Combined with the aforementioned analyses, these findings suggested that peiminine, a potential active constituent of ZBM–TBM, may contribute to anti‐BC effects through predicted interactions with hub genes such as AURKA.

G: GO enrichment analysis showing the major biological processes associated with the differentially expressed genes.

H: KEGG pathway enrichment analysis revealing the major signaling pathways associated with the differentially expressed genes.

## 4 Discussion

The occurrence and treatment of BC substantially impair patients'daily activities and markedly reduce quality of life. However, due to the lack of effective biomarkers and therapeutic targets, together with the existence of asymptomatic progression stages, BC is often diagnosed at relatively advanced stages, resulting in poor therapeutic efficacy and unfavorable prognosis [14]. ZBM–TBM has been reported to exhibit potential therapeutic effects against BC and may provide complementary benefits when combined with conventional therapies [15]. Owing to these properties, it has been widely used in the treatment of various tumors throughout its long history of clinical application [7]. Therefore, identifying effective biomarkers for BC and elucidating the interactions between therapeutic targets and drugs are of great importance [16].

In the present study, EPCAM was identified as a candidate key gene associated with BC through integration of multiple machine learning algorithms and network analyses. EPCAM consistently exhibited high stability across different machine learning algorithms and network analyses, thereby supporting its potential value as a candidate biomarker for BC. EPCAM, also known as CD326, is a transmembrane glycoprotein involved in calcium‐independent homophilic cell adhesion and participates in the regulation of signal transduction, cell migration, proliferation, and differentiation [17]. Previous studies have demonstrated that elevated EPCAM expression promotes tumor proliferation, maintenance of stemness phenotypes, and epithelial–mesenchymal transition in BC, while certain subpopulations may regulate the tumor microenvironment through immune‐inflammatory pathways [18, 19, 20]. GSEA further demonstrated that EPCAM was closely associated with processes including cell cycle regulation, DNA replication, and immune regulation, as well as tumor‐related signaling pathways such as AKT and transforming growth factor‐β signaling, which is consistent with its established roles in tumor proliferation and immune modulation [21]. Diagnostic and prognostic analyses further supported the potential clinical relevance of EPCAM, suggesting that EPCAM may have potential value for future risk assessment and biomarker development in BC. Previous studies have also reported that EPCAM has emerged as an important target for antitumor immunotherapy, with therapeutic strategies including the monoclonal antibody adecatumumab, the bispecific antibody catumaxomab, antibody–drug conjugates, and chimeric antigen receptor T‐cell therapy already entering early clinical investigation stages [22, 23, 24, 25].

Subsequently, LC–MS/MS analysis systematically identified 32 major active constituents in the ZBM–TBM herb pair, among which alkaloids represented the predominant constituent class, suggesting that these compounds may represent important chemical components contributing to the pharmacological properties of the herb pair. By constructing a drug–disease–target network, 33 overlapping targets and 10 hub genes were identified. Further enrichment analysis demonstrated that these genes were significantly enriched in immune‐related pathways as well as hypoxia‐inducible factor‐1, Notch, and other tumor‐related signaling pathways, suggesting that ZBM–TBM may potentially influence tumor‐associated biological processes through a coordinated multi‐target regulatory network. Immune infiltration analysis revealed that BC tissues exhibited a remodeled immune microenvironment characterized by reduced infiltration of B cells and CD4^+^memory T cells together with increased proportions of immunosuppressive Treg cells. Further correlation analysis demonstrated significant associations between hub genes and multiple immune cell subpopulations, suggesting that these genes may participate in regulation of the tumor immune microenvironment. These findings provide cellular‐level evidence supporting the potential involvement of immune regulation in the antitumor effects of ZBM–TBM.

Considering that the aforementioned analyses indicated potential diagnostic and prognostic relevance of EPCAM in BC, we further explored its potential associations within the molecular regulatory network. The present study demonstrated that although ZBM–TBM did not directly target EPCAM, regulatory network analysis revealed functional associations between EPCAM and DNMT1 as well as several hub genes (Figure 9). Among them, EPCAM expression was significantly positively correlated with DNMT1, and DNMT1 showed potential regulatory associations with EPCAM based on computational network analysis. These findings suggested that ZBM–TBM may be indirectly associated with EPCAM‐associated biological processes through hub gene‐related networks, thereby participating in BC‐related biological processes. Although direct upstream and downstream relationships among EPCAM, DNMT1, and AURKA have not been fully established, previous studies have independently demonstrated their important roles in breast cancer. EPCAM is involved in epithelial characteristics, tumor progression, and immune‐associated processes, whereas DNMT1 contributes to breast cancer development through epigenetic regulation and DNA methylation remodeling [26, 27]. AURKA functions as a critical cell‐cycle regulator and oncogenic kinase associated with proliferation and genomic stability [28]. Therefore, the associations identified in the present study may represent a coordinated molecular network involving epithelial phenotype regulation, epigenetic modification, and cell‐cycle control rather than a single linear regulatory axis.

At the molecular level, molecular docking and MD simulation analyses suggested that peiminine may represent a potential active constituent associated with the predicted anti‐BC effects of ZBM–TBM. Compared with other active constituents, peiminine exhibited markedly lower binding energies with multiple key targets and could stably occupy the active pockets of target proteins to form stable complexes (Figure 10). Among these targets, peiminine exhibited the strongest binding affinity toward AURKA and maintained stable complex conformations throughout the simulation process, suggesting favorable structural interaction characteristics. AURKA is a serine/threonine kinase closely associated with mitotic regulation and chromosomal stability, and its overexpression has been widely reported in various malignancies, including BC. Aberrant activation of AURKA contributes to uncontrolled cell proliferation, genomic instability, and malignant progression. Combined with the established biological functions of AURKA in G2/M transition and mitotic regulation, single‐cell virtual knockout analysis further suggested that computational perturbation of AURKA expression was associated with alterations in cell cycle‐related processes while also involving pathways associated with extracellular matrix remodeling and cell adhesion. These findings provide single‐cell computational evidence supporting the potential involvement of AURKA in tumor‐associated biological processes. The consistency between molecular interaction characteristics and computational cellular analyses suggested that AURKA may represent a candidate functional target associated with the potential biological effects of ZBM–TBM.

Consistent with these findings, the present study further confirmed that peiminine exhibited strong binding affinity and structural stability toward multiple hub targets, particularly AURKA. EPCAM also exhibited certain binding capabilities in molecular docking and MD simulation analyses. Combined with its position within the regulatory network and its functional associations with hub genes such as DNMT1, these findings suggested that EPCAM may be associated with ZBM–TBM‐related biological processes through hub gene‐associated networks. However, the current computational analyses cannot establish definitive upstream or downstream regulatory relationships among peiminine, hub genes, and EPCAM. Further experimental studies are required to validate the proposed molecular associations.

Compared with previous studies focusing mainly on the in vitro antiproliferative, pro‐apoptotic, or multidrug resistance‐reversing activities of Fritillaria alkaloids in individual cell lines and attributing these effects primarily to regulation of cell cycle‐, apoptosis‐, p53‐, or Bcl‐2‐related pathways [29, 30], the present study revealed from multiple perspectives that peiminine may act on a broader target network. Peiminine may potentially participate in BC‐related biological processes through predicted interactions with multiple hub genes and associated signaling pathways. These findings expand the current understanding of the molecular mechanisms of peiminine and provide new insights into the multi‐target synergistic regulatory characteristics of traditional Chinese medicine formulas.

Collectively, through integrated application of machine learning, network analysis, molecular simulation, and single‐cell computational analyses, the present study systematically explored the potentia role of EPCAM in BC and its potential associations with the antitumor mechanisms of ZBM–TBM. In addition, peiminine was predicted to be a potential core active constituent, whereas AURKA was identified as a potential functional target candidate associated with cell cycle regulation and tumor microenvironment‐related processes.

Nevertheless, several limitations should be acknowledged in the present study. First, although multiple public datasets and computational approaches were integrated, the current findings remain predictive and require further experimental validation. Second, the diagnostic and prognostic significance of EPCAM, the biological effects of peiminine, and the regulatory relationships involving AURKA and EPCAM should be confirmed through in vitro and in vivo studies. Third, molecular docking, molecular dynamics simulations, and single‐cell virtual knockout analyses provide mechanistic hypotheses rather than direct experimental evidence. Future studies incorporating cellular experiments, animal models, and clinical cohorts are warranted to validate these predicted mechanisms.

In future studies, emerging target discovery and validation technologies may provide further insights into the molecular mechanisms underlying the effects of the ZBM–TBM herb pair. Single‐cell omics approaches can be applied to characterize cell‐type‐specific responses and identify potential regulatory targets of active constituents within the tumor microenvironment [31]. In addition, proteomic approaches and advanced chemical biology strategies, such as PROTAC‐based target identification technologies, may facilitate the discovery and validation of direct protein targets and downstream signaling mechanisms of key active compounds [32]. These approaches, combined with in vitro and in vivo experimental models, may further clarify the molecular basis of the anti‐breast cancer effects of the ZBM–TBM herb pair.

## 5 Conclusion

In conclusion, the present study systematically explored the potential role of EPCAM in BC diagnosis, prognosis, and its possible association with the molecular mechanisms of the ZBM–TBM herb pair involving multiple hub genes. In addition, peiminine was predicted to be a potential active constituent and exhibited favorable predicted binding characteristics with multiple candidate targets, including AURKA, suggesting its possible involvement in the anti‐BC effects of ZBM–TBM. These findings provide new computational insights and theoretical directions for precision diagnosis and treatment research in BC, as well as for further investigation of traditional Chinese medicine‐derived antitumor candidates.

## Institutional Review Board Statement

Ethical review and approval is not applicable as this study does not involve human or animal subjects.

## Author Contributions

D.N., H.Z.Z., C.H.J., X.D.Z., and Guo Qing Zhao contributed the conception and design of the study. Quan Tang, He Li performed the experiments and interpreted the data. Quan Tang, X.D. Z. and Guo Qing Zhao revised the manuscript. All authors have read and agreed to the published version of the manuscript.

The research was supported by the following funding sources:1. National Natural Science Foundation of China (Grant No. 82274269); 2. Project supported by the Natural Science Foundation of Jiangsu Province, China (No. BK20240309); 3. Top Talent Support Program for young and middle‐aged people of Wuxi Health Committee (HB2023067) 4. Natural Science Foundation of Nanjing University of Chinese Medicine (XZR2023095).

## Conflicts of Interest

The authors declare no conflicts of interest.

## Supporting information


Supporting File

## Data Availability

Chemistry & amp; Biodiversity (CBDV). The datasets used and analyzed during the current study are publicly available. Gene expression data were obtained from the Gene Expression Omnibus (GEO, GSE15852), RNA‐seq and clinical data from The Cancer Genome Atlas (TCGA‐BRCA), and chemical structures from PubChem. Target predictions were sourced from the SwissTargetPrediction database. These datasets can be accessed from their respective repositories.
